# Differential glycosylation of envelope gp120 affects reactivity with HIV-1 specific antibodies

**DOI:** 10.1186/1471-2334-14-S2-P68

**Published:** 2014-05-23

**Authors:** Lydie Czernekova, Milan Raska, Zina Moldoveanu, Katerina Zachova, Stacy Hall, Dita Badalova, Alzbeta Krcmarska, Hana Synkova, Michael Hoelscher, Leonard Maboko, Rhubell Brown, Zdenek Novak, Jiri Mestecky, Jan Novak

**Affiliations:** 1 Department of Immunology, Palacky University, Olomouc, Czech Republic; 2 University of Alabama, Birmingham, Alabama, USA; 3 Clinic of the University of Munich, Munich, Germany; 4 NIMR-Mbeya Medical Research Program, Mbeya, Tanzania

## Introduction

Cellular entry of human immunodeficiency virus type 1 (HIV-1) depends on envelope glycoprotein (gp120/gp41) interactions with host-cell receptors. Approximately one-half of molecular mass of gp120 consists of N-glycans which may act as antigenic determinants as well as a shield against immune recognition. We have shown that glycosylation of subtype B HIV-1 gp120 varies according to the producing cell type and the differential glycosylation affects reactivities with serum antibodies of persons infected with HIV-1 subtype B. Here we studied reactivities of above proteins with panel of monoclonal gp120-specific broadly neutralizing antibodies and sera from persons infected with HIV-1 subtype A/C.

## Materials and methods

Recombinant gp120 produced in different cell lines (HEK 293T, Jurkat, RD, HepG2, and CHO) were tested as a native and after partial removal of N-glycans by PNGase F under native conditions. Several methods (ELISA, Cytometric Bead Array - CBA, SDS-PAGE with western blot, and dot blot) were used for determination and comparison of reactivities with monoclonal gp120-specific antibodies (268-D IV, F425 B4e8, 257-D IV, 447-52D, 19b, 2G12, and b12) or with sera of HIV-1 subtype A/C-infected persons.

## Results

After partial removal of N-glycans from gp120, the reactivity of most monoclonal antibodies increased, as did the reactivities of sera from HIV-1-infected persons. The largest increase in binding of polyclonal antibodies after PNGase F treatment was found for gp120 expressed in HEK 293T and CHO cell lines, common producers of recombinant proteins for vaccination purposes. Conversely, the gp120 produced by T cells (Jurkat) displayed the least increase in reactivity after partial deglycosylation.

## Conclusion

Changes in reactivity of selected monoclonal antibodies with native and PNGase F-treated proteins indicated that some glycans were resistant to deglycosylation and that this characteristic was dependent on producer cell type. Furthermore, CBA allowed more sensitive detection (about 40-times) of gp120-specific antibodies compared to ELISA.

Supported by CZ.1.07/2.3.00/20.0164 European Social Fund, UAB CFAR Developmental Grant (P30AI027767) and a Pilot Grant from UAB School of Medicine.

